# Impact of Covid-19 in pregnancy on mother’s psychological status and infant’s neurobehavioral development: a longitudinal cohort study in China

**DOI:** 10.1186/s12916-020-01825-1

**Published:** 2020-11-04

**Authors:** Yuanyuan Wang, Lian Chen, Tianchen Wu, Huifeng Shi, Qin Li, Hai Jiang, Danni Zheng, Xiaoli Wang, Yuan Wei, Yangyu Zhao, Jie Qiao

**Affiliations:** 1 Department of Obstetrics and Gynecology, Peking University Third Hospital, Beijing, 100191 China; 2 National Clinical Research Center for Obstetrical and Gynecology, Beijing, 100191 China; 3 National Center for Healthcare Quality Management in Obstetrics, Beijing, 100191 China; 4 Department of Maternal and Child Health, School of Public Health, Peking University, Beijing, 100191 China

**Keywords:** Covid-19, Maternal health, Child early development

## Abstract

**Background:**

Evidence concerning the long-term impact of Covid-19 in pregnancy on mother’s psychological disorder and infant’s developmental delay is unknown.

**Methods:**

This study is a longitudinal single-arm cohort study conducted in China between May 1 and July 31, 2020. Seventy-two pregnant patients with Covid-19 participated in follow-up surveys until 3 months after giving birth (57 cases) or having abortion (15 cases). We collected data from medical records regarding Covid-19, delivery or abortion, testing results of maternal and neonatal specimens, and questionnaires of quarantine, mother–baby separation, feeding, and measuring of mothers’ mental disorders and infants’ neurobehavioral disorders.

**Results:**

All cases infected in the first trimester and 1/3 of cases infected in the second trimester had an abortion to terminate the pregnancy. 22.2% of pregnant patients were suffering from post-traumatic stress disorder or depression at 3 months after delivery or induced abortion. Among 57 live births, only one neonate was positive of nucleic acid testing for throat swab, but negative in repeated tests subsequently. The median duration of mother–baby separation was 35 days (interquartile range 16 to 52 days). After the termination of maternal quarantine, 49.1% of mothers chose to prolong the mother–baby separation (median 8 days; IQR 5 to 23 days). The breastfeeding rate was 8.8% at 1 week after birth, 19.3% at the age of 1 month, and 36.8% at the age of 3 months, respectively. The proportion of “monitoring” and “risk” in the social–emotional developmental domain at the age of 3 months was 22.7% and 63.6%, respectively. After the adjustment of preterm, neonatal sex, admitted to NICU, and the mother’s Covid-19 condition, the negative associations were significantly identified (*p* < 0.05) between mother–baby separation days and three developmental domains: communication, gross motor, and personal–social.

**Conclusions:**

There is no definite evidence on vertical transmission of SARS-CoV-2. In addition to control infection risk, researchers and healthcare providers should pay more attention to maternal mental health and infant’s feeding, closeness with parents, and early development.

## Background

Coronavirus disease 2019 (Covid-19) is caused by severe acute respiratory syndrome coronavirus-2 (SARS-CoV-2). Another two notable coronavirus strains are severe acute respiratory syndrome coronavirus (SARS-CoV) and Middle East respiratory syndrome coronavirus (MERS-CoV). The Covid-19 pandemic has been the biggest global public health crisis in this century and will last a long time in the world. As of October 20, 2020, there were over 40 million confirmed cases worldwide, with over 1 million related deaths [1]. A line of literatures concerning pregnant patients infected with SARS-CoV-2 in different countries have been reported to describe their clinical features, potential risks for medical conditions (i.e., severe illness, ICU admission, and receipt of mechanical ventilation) or mother-to-baby vertical transmission, and maternal and neonatal outcomes [2–5]. The Covid-19 pandemic also would result in additional maternal and child deaths due to the potential disruption of health systems and decreased access to food, particularly in low-income and middle-income countries [6]. Besides such direct and indirect effects on the body’s physical health, however, evidence concerning the impact of Covid-19 in pregnancy on the neuropsychological function of pregnant patients and their offspring is unknown.

SARS-CoV-2 is a neuroinvasive virus capable of triggering a cytokine storm and hyperinflammation with potential effects on the central nervous system [7]. Such pathogenetical progress of the acute immune reaction, along with acute respiratory dysfunction, may cause the immediate and long-term consequences on cognitive and neuropsychological function [7, 8]. Furthermore, such emerging infectious diseases and the responding measures (i.e., isolation, quarantine, and social distancing) have tremendously impacted on people’s lifestyles and aroused psychological distress of high level [9–11]. Pregnancy is a special period in which women experience immunologic and physiologic changes that could increase their risk for psychological distress. Therefore, the pathophysiology of SARS-CoV-2 infection in pregnancy, along with the fear and uncertainty of short- and long-term effects on both themselves and their babies, would exacerbate psychological distress and mood alterations. Such multiple crises of SARS-CoV-2 infection in pregnancy may result in persistent impacts on mother’s psychological status as well as infant’s neurobehavioral development [12, 13]. Early mother–baby separation due to compulsory or voluntary quarantine may also have negative effects on infants’ feeding and early development [14, 15]. Both researchers and frontline health professionals call for more attention on the long-term impact of pregnant patients and their babies [16–18]. However, there has been no empirical evidence on the chronic effects of Covid-19 in pregnancy so far.

We conducted a cohort study on Covid-19 pregnant patients and their infants in China, based on a national epidemic reporting system established by the National Health Commission. This study aimed to evaluate the long-term impact of Covid-19 in pregnancy on mother’s psychological status and infant’s neurobehavioral development, to explore the association between mother–baby separation and child early development, and thereby to improve healthcare strategies on pregnant women, new mothers, and their babies during the Covid-19 pandemic.

## Methods

### Study design and participants

**Fig. 1:**
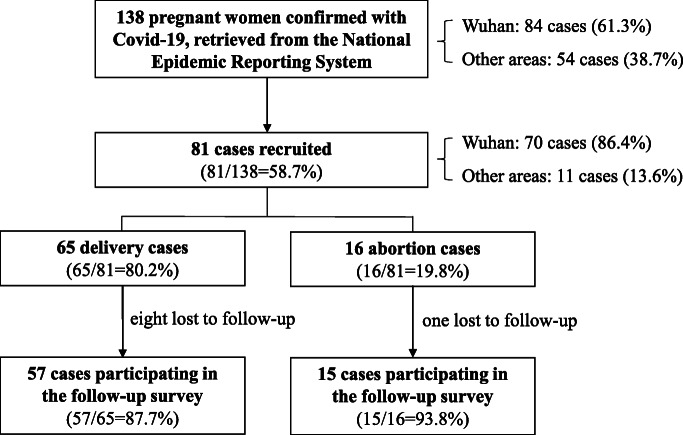
Flowchart of the cohort samples in this study

This study is a longitudinal single-arm cohort study conducted between May 1 and July 31, 2020. We retrieved a total of 138 pregnant women confirmed with Covid-19 from the National Epidemic Reporting System (NERS) established by the National Health Commission until April 30, 2020. Of these, 84 cases (61.3%) were from Wuhan, the hardest-hit area of the pandemic in China. All of those pregnant women were invited to participate in a follow-up survey until 3 months after delivery or abortion. The inclusion criteria include the following: (1) a confirmed case of Covid-19 was defined as a suspected case with a positive result on high-throughput sequencing or real-time reverse transcriptase-polymerase chain reaction (RT-PCR) assay of nasal and pharyngeal swab specimens; (2) a pregnant woman was diagnosed with Covid-19; (3) the onset of Covid-19 was in the pregnancy period; and (4) informed consent was obtained from the pregnant woman. The exclusion criteria include the following: (1) the onset of Covid-19 occurred before or after pregnancy and (2) those who lost to follow-up. Among these 138 pregnant patients across the country, 81 cases were recruited according to eligibility criteria with informed consent, including 65 delivery cases (80.2%) and 16 abortion cases (19.8%). As of July 31, 2020, we followed up 57 delivery cases and 15 abortion cases (Fig. 1). Comparison of maternal characteristics between 72 participants in this study and 138 pregnant patients reported in NERS can be seen in Additional file 1: Table S1.

### Procedures

For each participant, we collected data at four time points: (a) at the baseline when the pregnant woman was recruited, we collected data from medical records regarding the diagnosis, treatment, and outcomes of Covid-19; (b) at 1 week (7^+0~2^ days) after delivery or abortion, we collected data from medical records regarding hospitalized delivery, screening results of SARS-CoV-2 in different maternal and neonatal specimens (RT-PCR assay of neonatal throat swab, cord blood, amniotic fluid, breast milk, meconium, or placenta, and IgG and IgM in neonatal serum), postpartum care of mothers and neonates, or abortion procedure, and we also asked questions about the duration of quarantine, the status of mother–baby separation, and neonatal feeding; (c) at 1 month (28^+0~6^ days) after delivery or abortion, we followed up the duration of quarantine, the status of mother–baby separation, and infant feeding; and (d) at 3 months (90^+0~20^ days) after delivery or abortion, excepting for following up the duration of mother–baby separation and infant feeding, we measured the mother’s post-traumatic stress disorder (PTSD) symptoms using the PTSD Checklist-Civilian Version (PCL-C), the mother’s postpartum depression using the Edinburgh Postnatal Depression Scale (EPDS), and the infant’s neurobehavioral development using the Ages and Stages Questionnaires, third edition (ASQ-3) and the Ages and Stages Questionnaire: Social-Emotional, second edition (ASQ:SE-2). All of these questionnaires are valid and reliable to use in the Chinese populations [19–22]. All follow-up surveys were carried out via oral answers by telephone, or online questionnaire and photos of medical records by the Internet. All data were allowed to be collected prospectively or retrospectively, except that the measures of PCL-C, EPDS, ASQ-3, and ASQ:SE-2 were eligible to be done at 3 months after delivery or abortion.

PCL-C consists of 17 self-report items corresponding to the symptom criteria for PTSD in the Diagnostic and Statistical Manual of Psychiatric Disorders fourth edition (DSM-IV). Each item is rated on a 5-point scale, based on the extent to which the respondent has experienced specific symptoms in the past month. In this survey, the instructions provided to the respondents are as follows: “Referring to the current situation of Covid-19 outbreak, indicate how you feel for each of the following questions in the past month.” The total score ranges from 17 to 85 scores, with a higher score indicating a higher risk for PTSD. A total score of 17 to 37 scores is indicative of “without PTSD symptoms,” 38 to 49 scores indicating “potential risk of PTSD,” and 50–85 scores indicating “full PTSD diagnosis” [19]. EPDS consists of 10 self-report items on a 4-point scale, with a total score ranging from 0 to 30, based on how often the respondent has experienced specific symptoms in the past week. A total score of 0 to 9 scores is indicative of “without symptoms of postpartum depression,” 10 to 12 scores indicating “minor postpartum depression,” and 13–30 scores indicating “major postpartum depression” [20].

ASQ-3 and ASQ:SE-2 are two parent-rated screening tools for the delay of child early development at different ages and stages. In this study, we used the three 4-month subscale of ASQ-3 (designed for children aged 3 to 4 months) and the 6-month subscale of ASQ:SE-2 (designed for children aged 3 to 8 months) to evaluate the infant’s neurobehavioral development at 3 months of age. The ASQ-3 subscale contains 30 items divided into five developmental domains (six items per domain): communication, gross motor, fine motor, problem solving, and personal–social. The ASQ:SE-2 subscale contains 23 items for social–emotional. Each item is rated to a score of 0, 5, and 10, with a total score for each domain of ASQ-3 ranging from 0 to 60 scores and a total score for SE subscale ranging from 0 to 230. For each domain of ASQ-3 and ASQ:SE-2, a higher score indicates a higher developmental level for children at each month, and the developmental level can be divided into three zones: “normal,” “monitoring” for family guidance and re-screening, and “risk” for referral to intervention [23, 24].

### Statistical analysis

Continuous variables were described as medians with interquartile ranges (IQRs), and comparisons in different groups were performed using the Wilcoxon rank sum test. Categorical variables were described as counts with percentages, and comparisons in different groups were performed using Fisher’s exact test. We used scatter plots and Spearman’s rank correlation coefficient to assess the associations between mother–baby separation days and neurobehavioral development scores for each domain, which were further assessed by performing multiple linear regression modeling, with the adjustment of preterm (yes or no), neonatal sex (boy or girl), admitted to NICU (yes or no), and the mother’s Covid-19 condition in the pregnancy period (mild or severe). All statistical analyses were performed using SAS version 8.2. A two-tailed *p* value < 0.05 was considered as statistically significant.

All process of this study was reviewed and approved by the Peking University Third Hospital Medical Science Research Ethics Committee (No. IRB00006761-M2020127).

## Results

### Maternal characteristics and outcomes

Among 72 pregnant patients who participated in the follow-up survey (Table 1 and Additional file 1: Table S2), 13 cases (18.1%) were infected with SARS-CoV-2 in the first trimester, 6 cases (8.3%) in the second trimester, and 53 cases (73.6%) in the third trimester. A total of 57 pregnant patients (79.2%) gave a live birth, while 15 cases (20.8%) experienced an induced abortion but without any maternal indication. These abortion cases included 13 cases infected in the first trimester and 2 cases infected in the second trimester. In other words, all cases infected in the first trimester and 33.3% (2/6) of cases infected in the second trimester chose to have an abortion to terminate the pregnancy. Comparisons among sub-groups (delivery vs abortion, and three different trimesters) are also showed in Table 1 and Additional file 1: Table S2.

**Table 1:** Maternal characteristics and outcomes of pregnant women confirmed with Covid-19 in this study

Maternal characteristics/outcomes	Total (***n*** = 72)	Delivery group (***n*** = 57)	Abortion group (***n*** = 15)	***p*** value^**△**^
**Maternal and Covid-19 condition**				
Age (years, median (IQR))	31 (28, 34)	31 (28, 34)	32 (26, 35)	0.702
Region				
*Wuhan*	61 (84.7%)	48 (84.2%)	13 (86.7%)	1.000
*Other areas*	11 (15.3%)	9 (15.8%)	2 (13.3%)	
Infection period				
*1st trimester*	13 (18.1%)	0 (0.0%)	13 (86.7%)	**< 0.001**
*2nd trimester*	6 (8.3%)	4 (7.0%)	2 (13.3%)	
*3rd trimester*	53 (73.6%)	53 (93.0%)	0 (0.0%)	
Condition				
*Mild case*	63 (87.5%)	49 (86.0%)	14 (93.3%)	0.674
*Severe case*	9 (12.5%)	8 (14.0%)	1 (6.7%)	
Maternal complication				
*No*	59 (81.9%)	44 (77.2%)	15 (100.0%)	0.057
*Yes*	13 (18.1%)	13 (22.8%)	0 (0.0%)	
Outcome				
*Cure*	72 (100.0%)	57 (100.0%)	15 (100.0%)	–
*Death*	0 (0.0%)	0 (0.0%)	0 (0.0%)	
Hospital stay (days, median (IQR))	15 (9, 23)	15.5 (9, 23)	14.5 (7, 25)	0.987
Psychological condition at 3 months after delivery/abortion*				
PTSD (median (IQR))	24 (19, 32)	24 (19, 32)	27 (20, 36)	0.578
*Without PTSD symptoms*	52 (82.5%)	39 (81.3%)	13 (86.7%)	0.529
*Potential risk of PTSD*	6 (9.5%)	4 (8.3%)	2 (13.3%)	
*Full PTSD diagnosis*	5 (7.9%)	5 (10.4%)	0 (0.0%)	
EPDS (median (IQR))	3 (0, 6)	3 (0, 6)	4 (1, 7)	0.245
*Without symptoms of postpartum depression*	52 (82.5%)	39 (81.3%)	13 (86.7%)	0.712
*Minor postpartum depression*	4 (6.3%)	4 (8.3%)	0 (0.0%)	
*Major postpartum depression*	7 (11.1%)	5 (10.4%)	2 (13.3%)	

Percentages may not add to 100 because of rounding

*Among 57 cases in the delivery group, there were nine cases not responding to the questions on PTSD or EPDS

^△^The bold font in the column of “*p* value” indicates the significant difference (*p* < 0.05) between the delivery group and the abortion group

For the psychological condition at 3 months after delivery/abortion, 9.5% of pregnant women were at potential risk of PTSD, while 7.9% with full PTSD diagnosis; 6.3% of pregnant women had minor postpartum depression, while 11.1% having major postpartum depression. 22.2% (14/63) of pregnant women were suffering from PTSD or depression at 3 months after delivery or abortion. There was no significance in psychological conditions between the delivery group and the abortion group (Table 1).

### Infants’ characteristics and outcomes

A total of 57 live births born to pregnant patients in this study (Table 2), with 28 boys (49.1%) and 29 girls (50.9%). Of them, 18 (31.6%) were given birth at negative-pressure labor or operating room, 51 (89.5%) with cesarean section, 4 (7.0%) with low birth weight, 8 (14.0%) with preterm, 11 (19.3%) admitted to NICU (two cases with low birth weight, one case with pulmonary infection, eight cases for quarantine), 1 (1.8%) with congenital malformation (patent ductus arteriosus, PDA), and 1 (1.8%) having fetal distress and neonatal asphyxia. We also collected screening results of RT-PCR assay of neonatal throat swab (51 cases), cord blood (3 cases), amniotic fluid (2 cases), breast milk (12 cases), meconium (3 cases), and placenta (2 cases): one neonate with positive testing of throat swab (subsequent repeated tests of throat swabs and anal swabs were negative), and testing results of all other specimens were negative. There were 17 cases having testing of IgG and IgM in neonatal serum: 8 cases (47.1%) with IgG (+), including 3 cases (17.6%) with IgM (+). The neonate with positive testing of throat swab was a boy. His mother had positive testing of throat swab before the delivery, so the boy was immediately separated from his mother at birth. The first retrieval of his specimen on throat swab was taken at 36 h after birth, and the testing result of RT-PCR assay is positive. However, repeat retrieval and testing of throat swab at the third day after birth was negative. Subsequent repeated tests of throat swabs and anal swabs were also negative during the hospitalization. Finally, the boy was discharged from the hospital at the 17th day after birth.

**Table 2:** Characteristics and outcomes of infants born to pregnant women confirmed with Covid-19 in this study

Infants’ characteristics/outcomes	Total (***n*** = 57)	Boy (***n*** = 28)	Girl (***n*** = 29)	***p*** value^**△**^
**Perinatal conditions**				
Delivery unit				
Negative-pressure labor/operating room	18 (31.6%)	11 (39.3%)	7 (24.1%)	0.219
Isolated labor/operating room	39 (68.4%)	17 (60.7%)	22 (75.9%)	
Cesarean section	51 (89.5%)	26 (92.9%)	25 (86.2%)	0.670
Low birth weight (< 2500 g)	4 (7.0%)	1 (3.6%)	3 (10.3%)	0.611
Preterm (< 37 weeks)	8 (14.0%)	4 (14.3%)	4 (13.8%)	1.000
Congenital malformation	1 (1.8%)	1 (3.6%)	0 (0.0%)	1.000
Fetal distress	1 (1.8%)	0 (0.0%)	1 (3.4%)	1.000
Neonatal asphyxia	1 (1.8%)	0 (0.0%)	1 (3.4%)	1.000
Admitted to NICU	11 (19.3%)	2 (7.1%)	9 (31.0%)	**0.022**
Screening of SARS-CoV-2				
*Neonatal throat swab (+)*	1/51	1/23	0/28	0.451
*Cord blood (*+*)*	0/3	0/3	0/0	–
*Amniotic fluid (*+*)*	0/2	0/2	0/0	–
*Breast milk (*+*)*	0/12	0/9	0/3	–
*Meconium (*+*)*	0/3	0/3	0/0	–
*Placenta (*+*)*	0/2	0/2	0/0	–
*IgG in neonatal serum (*+*)*	8/17	5/12	3/5	0.620
*IgM in neonatal serum (*+*)*	3/17	2/12	1/5	1.000
**Mother–baby separation days**	35 (16, 52)	27 (16, 45)	38 (17, 55)	0.240
**Breastfeeding**				
At 1 week after birth	5 (8.8%)	2 (7.7%)	3 (10.7%)	1.000
At 1 month after birth	11 (19.3%)	6 (21.4%)	5 (17.9%)	0.689
At 3 months after birth	21 (36.8%)	12 (48.0%)	9 (33.3%)	0.355
Neurobehavioral development at 3 months after birth*				
Communication (median (IQR))	50 (40, 60)	50 (37.5, 55)	50 (40, 60)	0.380
“Monitoring”/“risk”	10 (19.2%)	6 (24.0%)	4 (14.8%)	0.492
Gross motor (median (IQR))	55 (45, 60)	55 (45, 60)	55 (45, 60)	0.931
“Monitoring”/“risk”	7 (13.5%)	5 (20.0%)	2 (7.4%)	0.241
Fine motor (median (IQR))	45 (40, 55)	45 (37.5, 55)	50 (40, 60)	0.498
“Monitoring”/“risk”	12 (23.1%)	6 (24.0%)	6 (22.2%)	0.879
Problem solving (median (IQR))	50 (40, 55)	50 (40, 55)	50 (40, 55)	0.941
“Monitoring”/“risk”	10 (19.2%)	5 (20.0%)	5 (18.5%)	1.000
Personal–social (median (IQR))	45 (40, 55)	45 (40, 55)	45 (35, 55)	0.643
“Monitoring”/“risk”	12 (23.1%)	5 (20.0%)	7 (25.9%)	0.746
Social–emotional (median (IQR))	25 (15, 35)	25 (20, 35)	15 (10, 35)	0.278
“Monitoring”/“risk”	38 (86.4%)	18 (94.7%)	20 (80.0%)	0.213

*Among the total of 57 cases, there were five cases (three boys and two girls) not responding to the questions on ASQ-3, and there were thirteen cases (nine boys and four girls) not responding to the questions on ASQ:SE-2

^△^The bold font in the column of “*p* value” indicates the significant difference (*p* < 0.05) between boys and girls

From the date of birth, the median duration of mother–baby separation was 35 days (IQR 16 to 52 days). After the termination of maternal quarantine, there were also 28 mothers (49.1%) choosing to prolong the mother–baby separation (median 8 days; IQR 5 to 23 days). The proportion of breastfeeding after birth was 8.8% at 1 week, 19.3% at 1 month, and 36.8% at 3 months, respectively. For neurobehavioral development at 3 months after birth, the total proportion of “monitoring” and “risk” at each domain of communication, gross motor, fine motor, problem solving, or personal–social was from 13.5 to 23.1%, while that proportion reached to 86.4% in the social–emotional domain of neurobehavioral development, including 22.7% at “monitoring” and 63.6% at “risk” (Table 1 and Additional file 1: Table S3).

Comparing with boys and girls, the proportion of girls admitted to NICU was higher than that of boys (31.0% vs 7.1%, *p* = 0.022), while no significance identified between two groups in other outcomes mentioned above. Detail data of infants’ outcomes can be seen in Table 1 and Additional file 1: Table S3.

### Association between mother–baby separation and neurobehavioral development

**Fig. 2:**
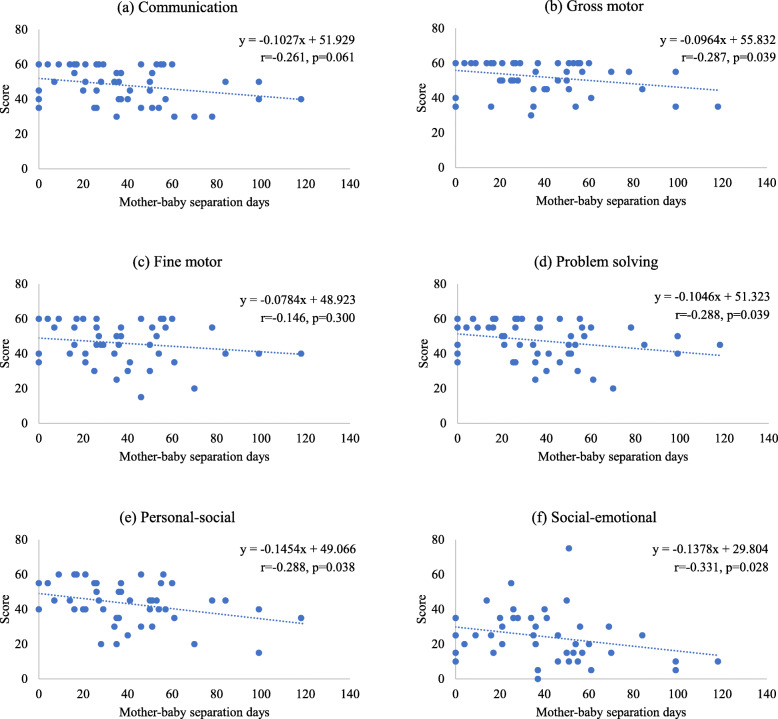
Association between mother–baby separation days and neurobehavioral development among infants born to Covid-19 pregnant patients

Figure 2 shows scatter plots, linear curves with equations, Spearman’s rank correlation coefficients, and *p* values between mother–baby separation days and scores in each domain of neurobehavioral development. Four domains of gross motor, problem solving, personal–social, and social–emotional were negatively linked with mother–baby separation days (*p* < 0.05). After the adjustment of preterm, neonatal sex, admitted to NICU, and the mother’s Covid-19 condition in the multiple linear regression modeling, the negative associations were significantly identified (*p* < 0.05) between mother–baby separation days and three domains: communication, gross motor, and personal–social (Table 3).

**Table 3:** Association between mother–baby separation days and neurobehavioral development: multiple linear regression

Dependent variable	Predictor: mother–baby separation days		
	***β*** coefficient (95% CI)	SE	***p*** value
Communication	− 0.133 (− 0.250, − 0.016)	0.058	**0.026**
Gross motor	− 0.112 (− 0.216, − 0.008)	0.052	**0.036**
Fine motor	− 0.112 (− 0.248, 0.024)	0.068	0.104
Problem solving	− 0.112 (− 0.233, 0.009)	0.060	0.068
Personal–social	− 0.188 (− 0.313, − 0.062)	0.062	**0.044**
Social–emotional	− 0.155 (− 0.340, 0.031)	0.092	0.099

In the multiple linear regression modeling, except for the predictor of “mother–baby separation days,” there are other predictors, including preterm (yes or no), neonatal sex (boy or girl), admitted to NICU (yes or no), and the mother’s Covid-19 condition in the pregnancy period (mild or severe)

## Discussion

Based on China’s National Epidemic Reporting System, we recruited 72 Covid-19 pregnant patients participating in this cohort study. We found that all pregnant patients infected in the first trimester and one third of those infected in the second trimester had an abortion but without any maternal indication and that the rate of cesarean section in the delivery group reached to nearly 90.0%. With the better understanding of Covid-19 in pregnancy and delivery, obstetricians and researchers have realized that pregnancy is not a poor prognostic factor in patients suffering from Covid-19 [25], the timing and mode of terminating pregnancy should be directed by obstetric factors and clinical urgency, and Covid-19 itself is not an indication for abortion or delivery [26, 27].

In this study, only one neonate, who had been reported in previous literature [28], was positive for throat swab RT-PCR testing at 36 h after birth, but negative in repeated tests subsequently. All other results of RT-PCR testing for maternal and neonatal specimens reported in this study were negative. Although eight neonates were positive for IgG and three of them were positive for IgM, sensitivity and specificity of IgG and IgM tests is a challenging way to diagnose congenital infections, less reliable than molecular diagnostic tests based on nucleic acid amplification and detection [29]. All currently published literatures of maternal–fetal vertical transmission were based on case report studies of one or few samples, but lacking evidence from large sample studies, let alone that cannot rule out the possibility of horizontal transmission immediately by the contact with infected mothers or healthcare personnel, or the maternal–fetal interface damaged due to other pathological factors [30–32]. Thus, we believe that there is still no definite evidence of vertical transmission of SARS-CoV-2.

Mother–baby separation is a problem worthy of great attention during the Covid-19 pandemic. In China, all cured patients are quarantined for 14 days to monitor the recurrence of Covid-19. Our study found that even after the end of quarantine, nearly a half of mothers continued to separate with their babies due to the fear of uncertain infection, along with a low proportion of breastfeeding in early months. Except for early cessation of breastfeeding, early mother–baby separation is also linked to negative effects on infant brain development, parent psychological well-being, and the parent–infant relationship [14, 33, 34]. Such adversity and stress during the prenatal and early childhood periods are associated with later impairments in learning, behavior, and both physical and mental well-being [35–37]. Compared with previous literatures, our study drawn the link more finely between mother–baby separation days and early developmental delay in many domains, such as communication, gross motor, problem solving, personal–social, and social–emotional, which makes the proof more precise. Although this study did not identify the association between mother–baby separation and maternal mental disorders (Additional file 1: Table S4), it figured out that over one fifth of pregnant patients suffered from PTSD or depression at 3 months after delivery or abortion. A recent study conducted in Turkey reported that 14.7% of pregnant patients had a risk for postpartum depression within 48 h after birth and the maternal–infant bonding status of women with depression was worse than that of women without depression [38]. Therefore, it is needed to promote health education, guidance, and monitoring of maternal mental health, breastfeeding, and parenting behaviors among pregnant women and their families, if necessary, timely referrals to psychiatrists or pediatricians for further interventions.

### Limitation of the study

The findings of this cohort study are subject to the limitation of small samples and lack of the control group. First, the source population size is small; there were one hundred and thirty-eight pregnant patients reported by the National Epidemic Reporting System. Second, the whole process of recruitment and follow-up surveys in this study were conducted by telephone and Internet, in which of the situations the objects’ participation and compliance would be not as satisfactory as face-to-face follow-ups; only seventy-two pregnant patients were included in this cohort study; the small sample size may lead to under-powered or false-negative results [39]. Third, without face-to-face approaches in field, it is too hard to set a high-quality control group. Furthermore, without a control group, the association between mother–baby separation and early developmental delay may be confounded by SARS-CoV-2 infection in pregnancy. Further research is needed to make up these flaws and gaps in the future.

## Conclusion

In summary, Covid-19 itself is not an indication for the timing and mode of terminating pregnancy. There is still lack of definite evidence on vertical transmission of SARS-CoV-2. In addition to control infection risk, researchers and healthcare providers should pay more attention to maternal mental health and infant’s feeding, closeness with parents, and early development. Therefore, we call for more large-sampling, high-quality designed, and long-term cohort studies, or even transnational studies are needed to follow up and evaluate the long-term effects on maternal and offspring health during the outbreak of Covid-19.

## Supplementary Information

**Additional file 1 : Table S1.** Comparison of maternal characteristics between participants in this study and all pregnant cases reported in NERS. **Table S2.** Maternal characteristics and outcomes of pregnant women confirmed with Covid-19 in three trimesters. **Table S3.** Infants’ neurobehavioral development at three months after delivery/abortion. **Table S4.** Spearman’s rank correlation coefficients of mother-baby separation, mothers’ mental disorders and infants’ neurobehavioral development.

## Data Availability

The datasets generated and/or analyzed during the current study are not publicly available due to local ownership of the data, but are accessible from the corresponding authors (Prof Jie Qiao, jie.qiao@263.net; Prof Yangyu Zhao, zhaoyangyu@bjmu.edu.cn; and Prof Yuan Wei, weiyuanbysy@163.com) on reasonable request.

## Abbreviations

ASQ: Ages and Stages Questionnaires
ASQ:SE: Ages and Stages Questionnaire: Social-Emotional
Covid-19: Coronavirus disease 2019
DSM-IV: Diagnostic and Statistical Manual of Psychiatric Disorders fourth edition
EPDS: Edinburgh Postnatal Depression Scale
ICU: Intensive care unit
MERS: Middle East respiratory syndrome
NERS: National Epidemic Reporting System
NICU: Neonatal intensive care unit
PCL-C: PTSD Checklist-Civilian Version
PTSD: Post-traumatic stress disorder
RT-PCR: Real-time reverse transcriptase-polymerase chain reaction
SARS: Severe acute respiratory syndrome
SARS-CoV-2: Severe acute respiratory syndrome coronavirus 2
