# Supplementary material for: Impact of Covid-19 in pregnancy on mother’s psychological status and infant’s neurobehavioral development: a longitudinal cohort study in China
Source: BMC Med. 2020 Nov 4;18:347. doi: 10.1186/s12916-020-01825-1 (PMC7609382; doi:10.1186/s12916-020-01825-1)
Supplement: Supplementary file 1 — Additional file 1 : Table S1. Comparison of maternal characteristics between participants in this study and all pregnant cases reported in NERS. Table S2. Maternal characteristics and outcomes of pregnant women confirmed with Covid-19 in three trimesters. Table S3. Infants’ neurobehavioral development at three months after delivery/abortion. Table S4. Spearman’s rank correlation coefficients of mother-baby separation, mothers’ mental disorders and infants’ neurobehavioral development. [file 12916_2020_1825_MOESM1_ESM.pdf]

Table S1. Comparison of maternal characteristics between participants in this study and all pregnant cases reported in NERS

Maternal characteristics/outcomes	Participants in this study (n=72)	All pregnant cases in the national epidemic reporting system (n=138)	P value△
Age (years, mean (SD))	31 (28, 34)	31 (27, 34)	0.734
Region			
Wuhan	61 (84.7%)	84 (61.3%)	**<0.001**
Other areas	11 (15.3%)	54 (38.7%)	
Infection period			
1st trimester	22 (30.6%)	28 (20.3)	**0.014**
2nd trimester	6 (8.3%)	31 (22.5)	
3rd trimester	53 (73.6%)	79 (57.2)	
Condition			
Mild case	63 (87.5%)	121 (87.7%)	0.970
Severe case	9 (12.5%)	17 (12.3%)	
Maternal complication			
No	59 (81.9%)	130 (94.2%)	**0.005**
Yes	13 (18.1%)	8 (5.8%)	
Outcome			
Cure	72 (100.0%)	137 (99.3%)	1.000
Death	0 (0.0%)	1 (0.7%)	
Hospital stay (days, median (IQR))	15 (9, 23)	14 (9, 21)	0.626

△ **The BLOD Font** in the column of *P value* indicates the significant difference (P<0.05) between two groups.

Table S2. Maternal characteristics and outcomes of pregnant women confirmed with Covid-19 in three trimesters

Maternal characteristics/outcomes	Total (n=72)	1st trimester (n=13)	2nd trimester (n=6)	3rd trimester (n=53)	P value△
Covid-19 condition					
Mild case	63 (87.5%)	12 (92.3%)	6 (100.0%)	45 (84.9%)	0.717
Severe case	9 (12.5%)	1 (7.7%)	0 (0.0%)	8 (15.1%)	
Maternal complication					
No	59 (81.9%)	13 (100.0%)	5 (83.3%)	41 (77.4%)	0.167
Yes	13 (18.1%)	0 (0.0%)	1 (16.7%)	12 (22.6%)	
Hospital stay (days, median (IQR))	15 (9, 23)	14 (7, 16.5)	15.5 (12, 27.5)	16.5 (9, 23)	0.570
Pregnancy outcome					
Delivery	57 (79.2%)	0 (0.0%)	4 (66.7%)	53 (100.0%)	**<0.001**
Abortion	15 (20.8%)	13 (100.0%)	2 (33.3%)	0 (0.0%)	
Preterm					
No	49/57 (86.0%)	—	4/4 (100.0%)	45/53 (84.9%)	0.627
Yes	8/57 (14.0%)	—	0/4 (0.0%)	8/53 (15.1%)	

△ **The BLOD Font** in the column of *P value* indicates the significant difference (P<0.05) among three groups.

Table S3. Infants' neurobehavioral development at three months after delivery/abortion

Infants' characteristics	Total (n=57)	Boy (n=28)	Girl (n=29)	P value△
Communication				
Normal	42 (80.8%)	19 (76.0%)	23 (85.2%)	0.492
Monitoring	10 (19.2%)	6 (24.0%)	4 (14.8%)	
Risk	0 (0.0%)	0 (0.0%)	0 (0.0%)	
Gross motor				
Normal	45 (86.5%)	20 (80.0%)	25 (92.6%)	0.241
Monitoring	7 (13.5%)	5 (20.0%)	2 (7.4%)	
Risk	0 (0.0%)	0 (0.0%)	0 (0.0%)	
Fine motor				
Normal	40 (76.9%)	19 (76.0%)	21 (77.8%)	0.891
Monitoring	9 (17.3%)	4 (16.0%)	5 (18.5%)	
Risk	3 (5.8%)	2 (8.0%)	1 (3.7%)	
Problem solving				
Normal	42 (80.8%)	20 (80.0%)	22 (81.5%)	0.882
Monitoring	7 (13.5%)	4 (16.0%)	3 (11.1%)	
Risk	3 (5.8%)	1 (4.0%)	2 (7.4%)	
Personal-social				
Normal	40 (76.9%)	20 (80.0%)	20 (74.1%)	0.455
Monitoring	7 (13.5%)	4 (16.0%)	3 (11.1%)	
Risk	5 (9.6%)	1 (4.0%)	4 (14.8%)	
Social-emotional				
Normal	6 (13.6%)	1 (5.3%)	5 (20.0%)	0.103
Monitoring	10 (22.7%)	7 (36.8%)	3 (12.0%)	
Risk	28 (63.6%)	11 (57.9%)	17 (68.0%)	

Percentages may not add to 100 because rounding.

* Among the total of 57 cases, there were five cases (three boys and two girls) not responding to the questions on ASQ-3; and there were thirteen cases (nine boys and four girls) not responding to the questions on ASQ:SE-2.

△ **The BLOD Font** in the column of *P value* indicates the significant difference (P<0.05) among three groups.

Table S4. Spearman's rank correlation coefficients of mother-baby separation, mothers' mental disorders and infants' neurobehavioral development

	1	2	3	4	5	6	7	8	9
**1. Mother-baby separation (days)**	1								
**2. PTSD (scores)**	0.083	1							
**3. EPDS (scores)**	0.121	**0.796**	1						
**4. Communication (scores)**	-0.261	-0.126	-0.248	1					
**5. Gross motor (scores)**	**-0.287**	-0.057	-0.250	**0.536**	1				
**6. Fine motor (scores)**	-0.146	0.026	0.032	**0.572**	**0.598**	1			
**7. Problem solving (scores)**	**-0.288**	-0.152	-0.164	**0.619**	**0.687**	**0.697**	1		
**8. Personal-social (scores)**	**-0.288**	0.032	-0.077	**0.523**	**0.648**	**0.634**	**0.655**	1	
**9. Social-emotional (scores)**	**-0.331**	0.055	0.071	-0.142	**-0.316**	-0.293	-0.241	-0.042	1

△ **The BLOD Font** indicates $P<0.05$.
